# Supplementary figures and images for: Investigating the influence of mtDNA and nuclear encoded mitochondrial variants on high intensity interval training outcomes
Source: Sci Rep. 2020 Jul 6;10:11089. doi: 10.1038/s41598-020-67870-1 (PMC7338527; doi:10.1038/s41598-020-67870-1)

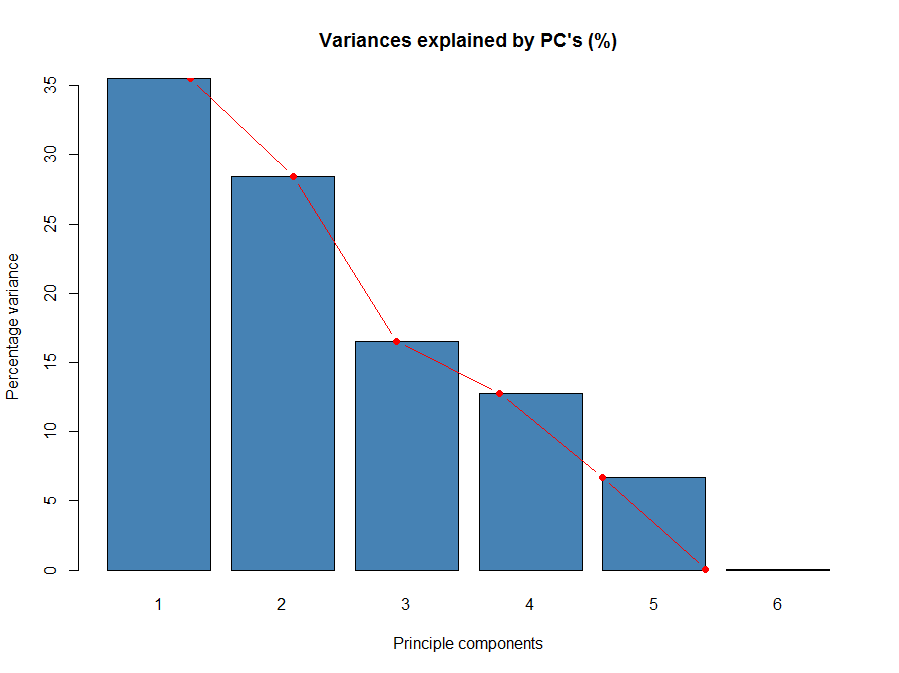

Supplement: Supplementary file 1 — Supplementary file1 (PNG 8 kb) [file 41598_2020_67870_MOESM1_ESM.png]

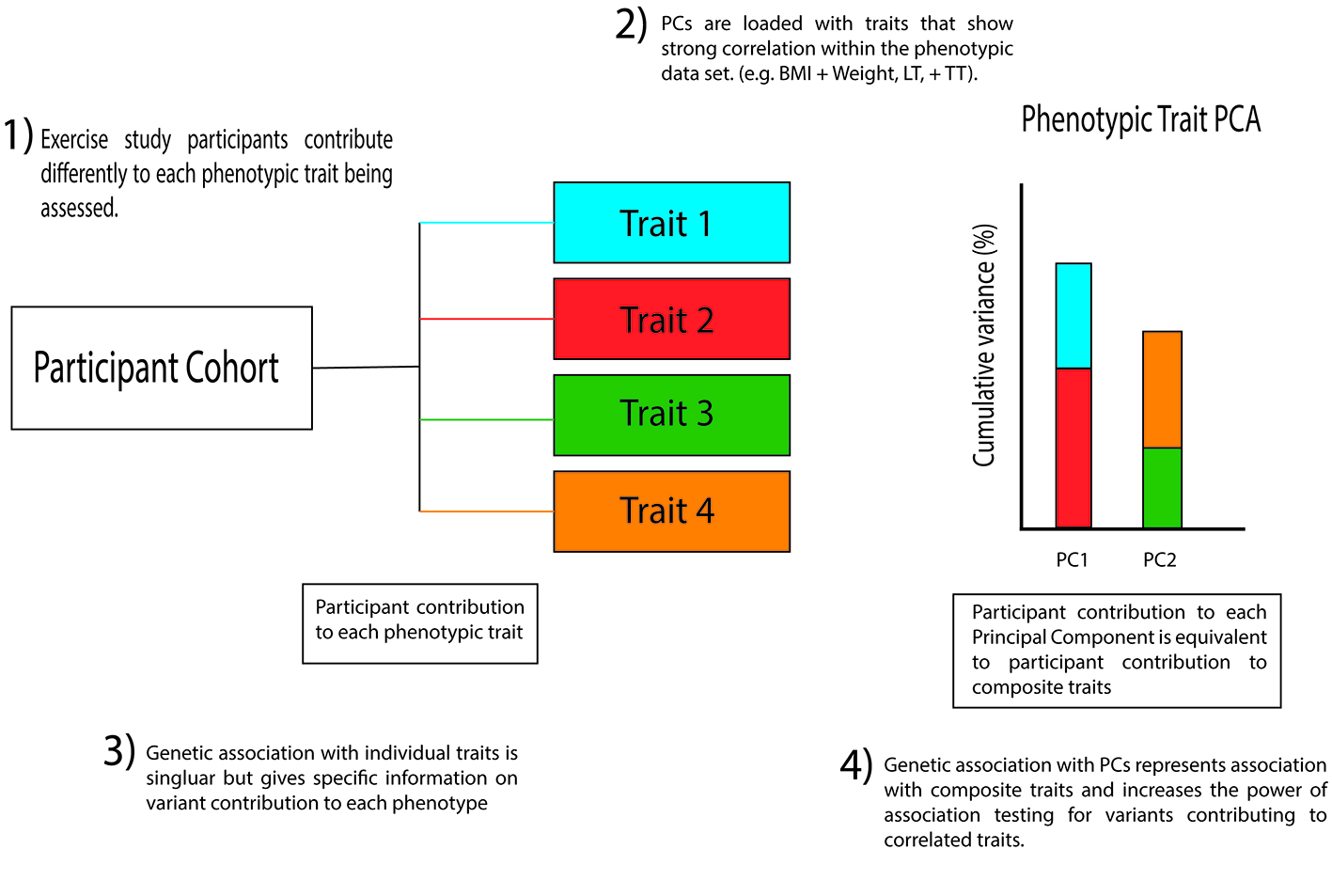

Supplement: Supplementary file 2 — Supplementary file2 [file 41598_2020_67870_MOESM2_ESM.tif]
